# Gut Microbiota Dysbiosis in the Development and Progression of Gastric Cancer

**DOI:** 10.1155/2022/9971619

**Published:** 2022-08-28

**Authors:** Yingying Miao, Hui Tang, Qizhi Zhai, Lu Liu, Lu Xia, Wenhan Wu, Yue Xu, Jianning Wang

**Affiliations:** ^1^Department of Gastroenterology, The Affiliated Jiangning Hospital of Nanjing Medical University, Nanjing 211199, China; ^2^Shanghai Biotecan Pharmaceuticals Co., Ltd., Shanghai 201204, China; ^3^Shanghai Zhangjiang Institute of Medical Innovation, Shanghai 201204, China

## Abstract

**Objectives:**

This study aims to explore gut microbiota dysbiosis in the histological stages of gastric cancer (GC).

**Methods:**

Feces samples and clinical characteristics were collected from patients with different stages of GC, including 15 superficial gastritis (SG), 13 atrophic gastritis (AG), 8 gastric mucosal atypical hyperplasia (GMAH), and 15 advanced GC cases. The diversity and composition of gut microbiota among the four groups were determined by sequencing the V4 region of bacterial 16S rRNA genes.

**Results:**

Reduced gut microbial alpha diversity and altered dissimilarity of the microbial community structure were found among the four groups. In addition, 18 species, 6 species, 6 species, and 16 species of bacteria were enriched in the SG, AG, GMAH, and GC groups, respectively, using the linear discriminant analysis (LDA) effect size (LEfSe) analyses. Besides, we found that two new genera, *Scardovia* and *Halomonas*, are associated with GC and the metabolic pathways of Genetic information processing and Circulatory System were more abundant in the GC group compared with noncancer groups.

**Conclusions:**

We identified differences in microbial compositional changes across stages of GC. Six genera and two metabolic pathways were more abundant in the GC group than noncancer groups, suggesting that these findings may contribute to the therapy strategies in GC in the near feature.

## 1. Introduction

Gastric cancer (GC) is the fifth common malignant tumor in the world and around 1 million new patients were diagnosed in 2018 [1]. GC usually develops through multistep processes of histological progression from atrophic gastritis (AG) progresses to intestinal metaplasia (IM), followed by gastric mucosal atypical hyperplasia (GMAH) and finally GC [2]. GC is a complex disease which involves many factors, such as host genetics, environmental factors, and microbial factors [3].

Furthermore, *Helicobacter pylori* (HP) infection is known as the major risk factor for the development of GC [4], which stimulates immune and inflammatory responses that reduce acid secretion, thus resulting in more gastric bacterial colonization [5], and can lead to the alternations in the composition of gut microbiota [6]. HP is classified as a class 1 carcinogen by the World Health Organization [7]. Recent studies reported that the dysbiosis of gut microbiota can lead to many diseases [8], including inflammatory bowel disease, diabetes, obesity, metabolic syndrome, and cardiovascular disease [9], as well as cancers like GC [10]. The gut microbiota plays an important role in human health through regulating host immune responses, energy metabolism, and eliminating pathogen and oncogenesis [11]. Researches revealed that the composition of the gastric microbiota is affected by many factors including HP, health status, dietary habits, medication use, age, and operation treatment [12, 13]. Recently, Zhang et al. performed 16S rRNA gene analysis in gastric mucosal specimens for 47 patients with SG, AG, gastric intraepithelial neoplasia (GIN), or GC and found that *Parvimonas*, *Eikenella*, *Prevotella-2*, *Kroppenstedtia*, *Lentibacillus*, and *Oceanobacillus* were enriched in the GC patients [14].

However, the distribution of gut microbiota in the progression of GC development remains unknown. Thus, it is urgent to reveal the roles of gut microbiota dysbiosis in the progress of GC pathogenesis and to develop potential prevention and treatment strategies in GC.

In the present study, we applied 16S rRNA gene sequencing to characterize the changes in the gut microbial composition and ecology in order to explore their roles in the development and progression of GC, sequentially from SG to AG, GMAH, and GC.

## 2. Materials and Methods

### 2.1. Patients Recruitment

Totally, 15 SG, 13 AG, 8 GMAH, and 15 GC patients were recruited from the Affiliated Jiangning Hospital of Nanjing Medical University (Nanjing, China) from May 2020 to June 2021. For pathological diagnosis, SG and AG specimens were obtained by upper gastroenterology endoscopic examination. GMAH specimens were obtained by endoscopic submucosal dissection (ESD) as described [14] and GC specimens were obtained by surgery or gastroenterology endoscopic examination. In addition, 7 clinical characteristics of all subjects were collected such as age, sex, Body Mass Index (BMI), smoking or drinking status, HP infection status, and Family history of GC. BMI was measured in kg/m^2^, and was defined as underweight <19, normal = 19–25 and overweight >25.

All procedures were approved by the ethical standards of the Clinical Research Ethics Committee of the Affiliated Jiangning Hospital of Nanjing Medical University, and the written informed consents were obtained from all participants who participated in the study.

### 2.2. Fecal DNA Extraction and 16s rRNA Gene Sequencing

Total 51 fresh feces samples were collected and then stored at −20°C for further analysis. Microbial DNA was extracted from 200 mg fecal sample and purified using the QIAamp PowerFecal Pro DNA Kit (QIAGEN) according to a previous study [15]. Then DNA was amplified using universal primers 515F 5′-GTGYCAGCMGCCGCGGTA-3′ and 806R 5′-GGACTACNVGGGTWTCTAAT-3′, which target the 16S rRNA genes V4 hypervariable regions. PCR was performed by Veriti™ 96-Well Thermal Cycler PCR system (Thermo Fisher Scientific) and was run by the following program: 95°C for 3 min, followed by 21 cycles of 95°C for 30 s, 56°C for 30 s, 72°C for 30 s, and 72°C for 5 min for a final extension. The resulting amplicon library was performed at Shanghai Biotecan Pharmaceuticals Co., Ltd. (Shanghai, China), using the Illumina Novaseq 6000 Sequencing system (Illumina, USA). All the experimental protocols were performed in accordance with the relevant guidelines and regulations.

### 2.3. Data Analysis

Sequences data were performed by mothur software package (v.1.39.5) and were assigned to the 97% similarity of operational taxonomic units (OTUs), which was compared by Greengenes database, performed by the Quantitative Insights into Microbial Ecology (QIIME) software package [16]. The alpha diversity was used to calculate ACE, Chao1, Shannon, and Simpson indexes in the mothur. The beta diversity was assessed by Principal Coordinate Analysis (PCoA) on a Bray-Curtis distance matrix, unweighted UniFrac distances, and weighted UniFrac distances in the QIIME software. The linear discriminant analysis (LDA) effect size (LEfSe) analyses were used to identify the relative abundances of taxa, using the absolute LDA score (log10) >3.0 with *p* < 0.05. The Kyoto Encyclopedia of Genes and Genomes (KEGG) [17] pathways were categorized using Phylogenetic Investigation of Communities by Reconstruction of Unobserved States (PiCRUSt) and were imported into STAMP (v.2.1.3) for visualization.

### 2.4. Statistical Analysis

The data are shown as the mean ± standard deviation. Differentially abundant bacterial taxa were identified by using the Wilcoxon rank-sum test (between two groups) or the Kruskal-Wallis test (more than two groups) in *R* studio (v.3.6.1). Clinical data analyses in the four groups were conducted by one-way ANOVA test using Prism version 6.0 (GraphPad, San Diego, CA, USA) or chi-squared test using SPSS 19.0. *p* value <0.05 was considered significant in statistics.

## 3. Results

### 3.1. Characteristics of the Subjects

Seven clinical features of all subjects are shown in Table 1, including sex, age, BMI, family history of GC, smoking or drinking status, and HP infection status. The results indicated that age (*p* < 0.0001), BMI (*p*=0.0011), smoking status (*p*=0.016), drinking status (*p*=0.02), and HP infection status (*p*=0.004) were significantly different among the four groups, except sex and family history of GC. Moreover, the patients in GMAH and GC groups were older than those in the SG and AG groups.

### 3.2. Characteristics of the 16s rRNA Gene Sequencing Results

**Figure 1:**
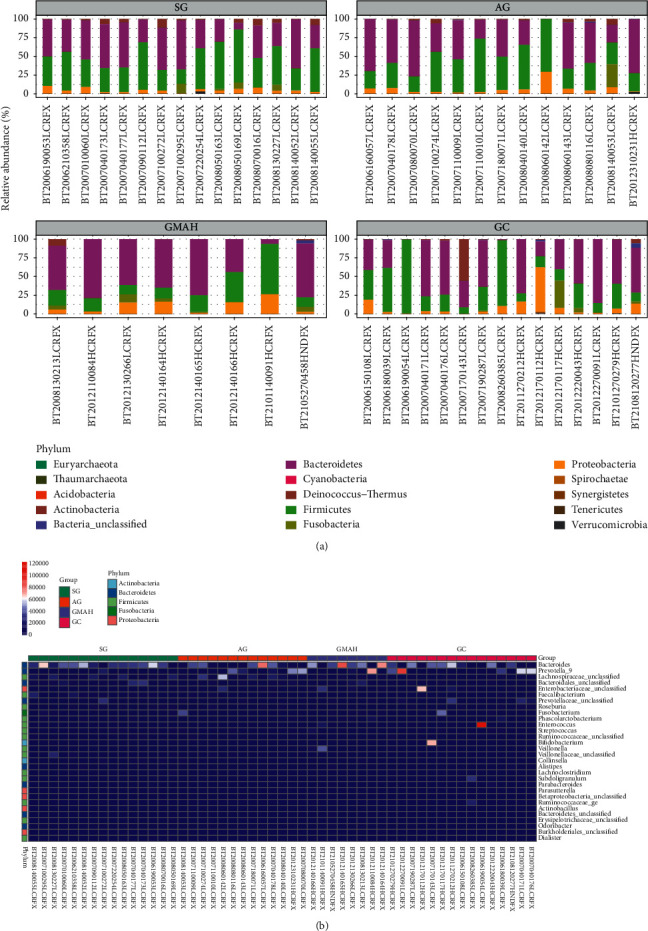
Characteristics of the gut microbiota composition among the four groups. (a) The distributions of the relative abundance of phylum level in the four groups. (b) The top 30 genera in the four groups were listed by heat map. SG: superficial gastritis; AG: atrophic gastritis; GMAH: gastric mucosal atypical hyperplasia; GC: gastric cancer.

To characterize the gut microbiota associated with different stages of GC, 16S rRNA genes sequencing was applied to 51 fecal samples collected from SG, AG, GMAH, and GC groups. A total of 2414, 2860, 1166, and 2531 OTUs (97% similarity) were obtained from SG, AG, GMAH, and GC groups, respectively (Table S1). The top 3 dominant bacterial phyla of each group were Bacteroidetes, Firmicutes, and Proteobacteria (Figure 1(a)). In addition, a heat map was constructed to describe the top 30 genera in each group (Figure 1(b)), in which *Bacteroides*, *Prevotella_9,* and Lachnospiraceae_unclassified are the top 3 genera.

### 3.3. Characteristics of Gut Microbiota Alpha Diversity and Beta Diversity

**Figure 2:**
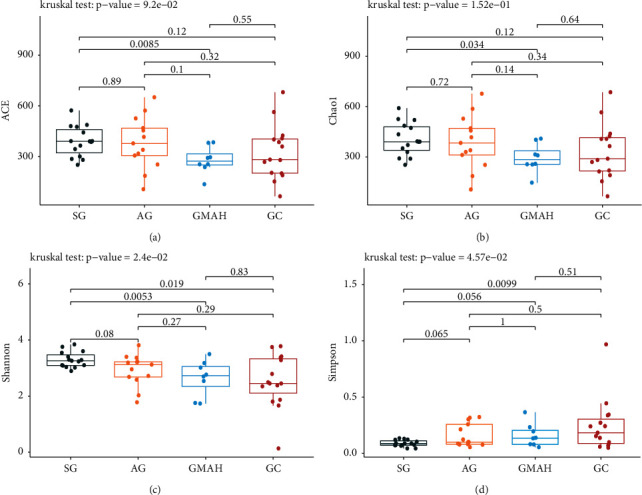
Comparison of the microbiota alpha diversity among the four groups. The community richness index ACE (a) and chao1 (b) and the community diversity index Shannon (c) and Simpson (d) were used to assess the alpha diversity. ^*∗*^*p* < 0.05. SG: superficial gastritis; AG: atrophic gastritis; GMAH: gastric mucosal atypical hyperplasia; GC: gastric cancer.

**Figure 3:**
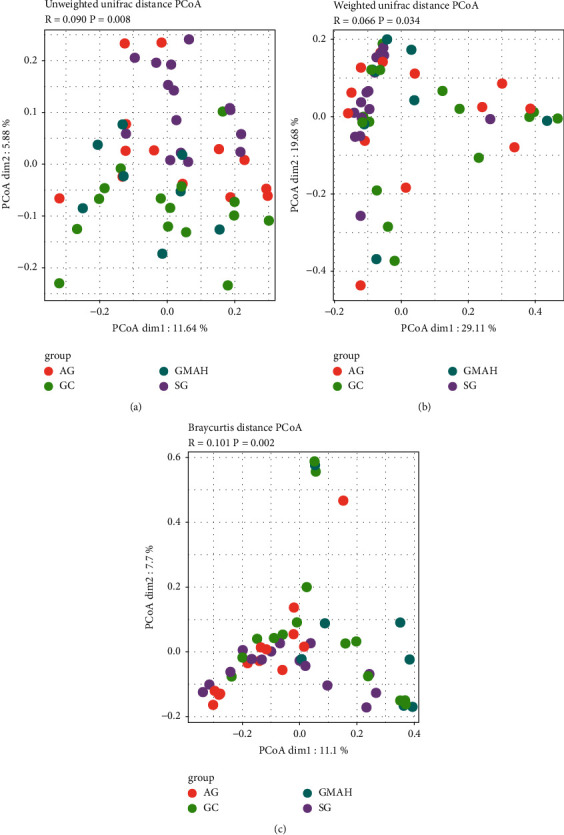
Comparison of the microbiota beta diversity among the four groups. The PCoA was used to evaluate the beta diversity by unweighted UniFrac distances (a), weighted UniFrac distances (b), and Bray-Curtis distance matrix (c). ^*∗*^*p* < 0.05. SG: superficial gastritis; AG: atrophic gastritis; GMAH: gastric mucosal atypical hyperplasia; GC: gastric cancer.

Alpha diversity and beta diversity were used to assess the gut microbiota dysbiosis among the four groups. The community richness (ACE and Chao1 indexes) and the community diversity (Shannon and Simpson indexes) were used to assess alpha diversity. Compared with SG, the ACE and Chao1 indexes were significantly reduced in the GMAH group (Figures 2(a) and 2(b)) and the Shannon indexes were significantly lower in both GMAH and GC groups, while the Simpson index was only significantly higher in the GC group (Figures 2(c) and 2(d)). The results suggest that noticeable changes of richness and diversity were observed in the gut microbiota profile of the GMAH and the GC groups. In addition, the PCoA of Bray-Curtis distance matrix, unweighted UniFrac distances, and weighted UniFrac distances were performed to evaluate the beta diversity, which was used to calculate the composition of gut microbial dissimilarity. The PCoA diagrams show significant separation of the gut microbiota among the four groups (Figures 3(a)–3(c)).

### 3.4. Characteristics of the Microbial Structure Profiles

**Figure 4:**
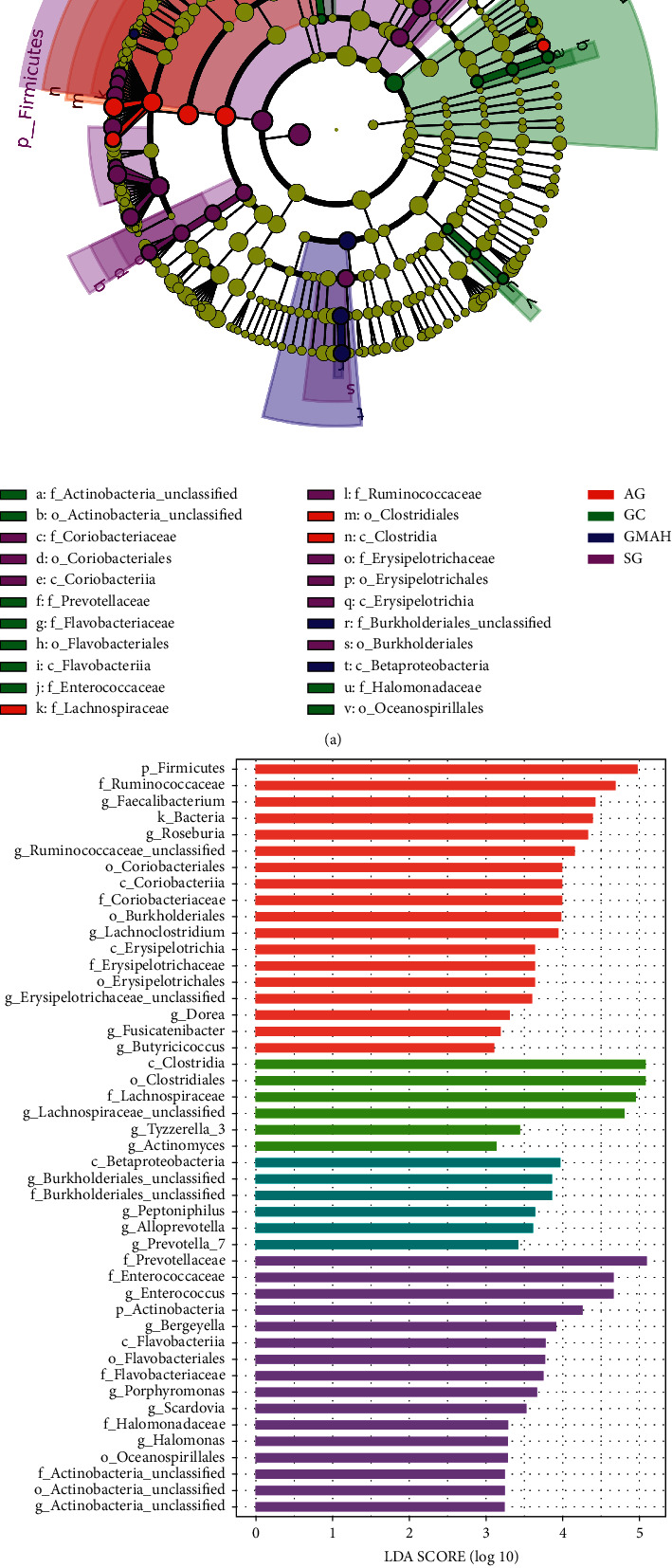
Comparing the distributions of the gut microbiota structure and composition among the four groups. (a) The cladogram illustrates the phylogenetic distribution of microbial lineages among the four groups. Differently abundant microbiota is listed and marked by different color (b). SG: superficial gastritis; AG: atrophic gastritis; GMAH: gastric mucosal atypical hyperplasia; GC: gastric cancer; p: phylum; c: class; o: order; f: family; g: genus.

The differing microbial communities were further confirmed by LEfSe analyses, which used LDA to identify significantly abundant bacteria among the four groups (LDA score >3.0 with *p* < 0.05). The resulting cladogram showed the most significant difference at taxonomic levels among the four groups. The circle size symbolizes the abundance of certain bacteria (Figure 4(a)).

Totally, 46 significant phylotypes were identified, in which 18, 6, 6, and 16 species of bacteria were abundant in the SG, AG, GMAH, and GC groups, respectively (Figure 4(b)). At the genus level, 8 genera (*Dorea* (*p*=0.037), Erysipelotrichaceae_unclassified (*p*=0.0151), Ruminococcaceae_unclassified (*p*=0.0259), *Fusicatenibacter* (*p*=0.00357), *Faecalibacterium* (*p*=0.00895), *Roseburia* (*p*=0.0318), *Lachnoclostridium* (*p*=0.000259), and *Butyricicoccus* (*p*=0.0133)) were significantly enriched in the SG group; 3 genera (*Tyzzerella_3* (*p*=0.0199), *Actinomyces* (*p*=0.0455), and Lachnospiraceae_unclassified (*p*=1.38*e*^−5^)) were significantly enriched in the AG group; 4 genera (Burkholderiales_unclassified (*p*=0.0378), *Peptoniphilus* (*p*=0.0204), *Alloprevotella* (*p*=0.00581), and *Prevotella_7* (*p*=0.00740)) were significantly enriched in the GMAH group; and 6 genera (*Porphyromonas* (*p*=0.0486), *Scardovia* (*p*=0.0170), *Halomonas* (*p*=0.0171), Actinobacteria_unclassified (*p*=0.00562), *Bergeyella* (*p*=0.0170), and *Enterococcus* (*p*=0.0474)) were significantly enriched in the GC group.

### 3.5. Analysis of the Correlation between Clinical Data and Gut Microbiota

Through the LEfSe analyses, 21 bacterial genera significantly differed among the four groups. To investigate the interactions between gut microbiota and the clinical status of all subjects, we identified the statistical correlations between the 21 genera and the 7 clinical features.

**Figure 5:**
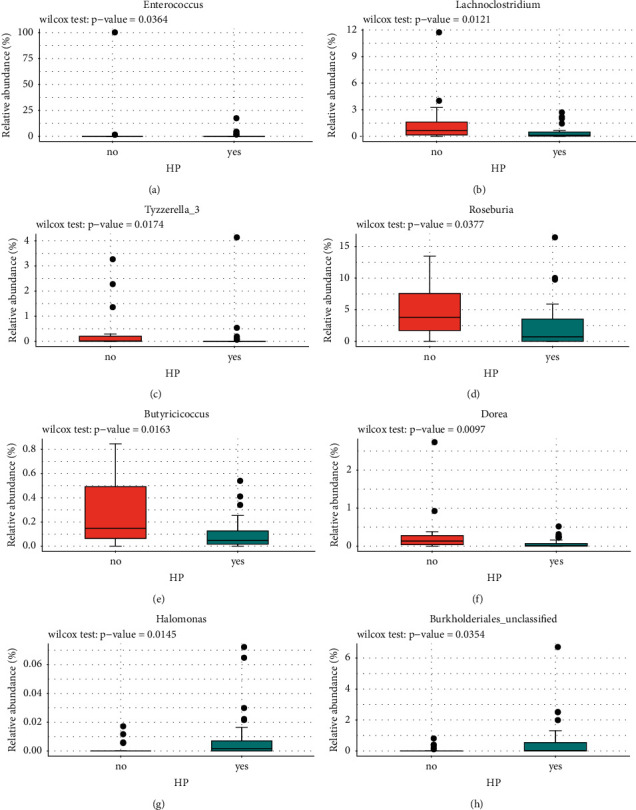
Comparison of the abundance of the 21 LDA-differentiated genera between non-HP and HP infection groups. *Enterococcus* (a), Lachnospiraceae_unclassified (b), *Tyzzerella_3* (c), *Roseburia* (d), *Butyricicoccus* (e), and *Dorea* (f) were less abundant in the HP infection group, while the levels of *Halomonas* (g) and Burkholderiales_unclassified (h) were significantly higher in the HP infection group. ^*∗*^*p* < 0.05. HP: *Helicobacter pylori*.

**Figure 6:**
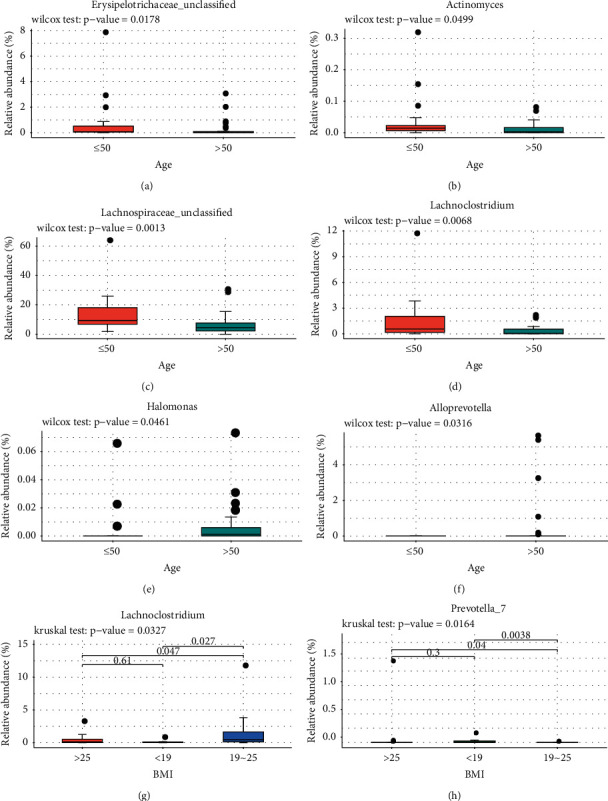
Comparison of the abundance of the 21 LDA-differentiated genera between ≤50 age and >50 age groups. The levels of Erysipelotrichaceae_unclassified (a), *Actinomyces* (b), Lachnospiraceae_unclassified (c), and *Lachnoclostridium* (d) genus were lower in the >50 age group, while *Alloprevotella* (e) and *Halomonas* (f) were significantly higher in the ≤50 age group. Moreover, *Lachnoclostridium* (g) and *Prevotella_7* (h) were significantly different among underweight, normal, and overweight BMI groups. ^*∗*^*p* < 0.05. BMI: body mass index.

To be specific, we found that the abundances of *Enterococcus, Lachnoclostridium, Tyzzerella_3, Roseburia, Butyricicoccus,* and *Dorea* were significantly lower in the HP infection group than the non-HP infection group (Figures 5(a)–5(f)). The abundances of *Halomonas* and Burkholderiales_unclassified were noticeably higher in the HP infection group than the non-HP infection group (Figures 5(g) and 5(h)). The abundances of Erysipelotrichaceae_unclassified*, Actinomyces,* Lachnospiraceae_unclassified, and *Lachnoclostridium* genus were lower in the older age group (>50) (Figures 6(a)–6(d)), while *Halomonas* and *Alloprevotella* were significantly enriched in the older age group (Figures 6(e) and 6(f)). In addition, the abundances of *Lachnoclostridium* and *Prevotella_7* were significantly different among underweight, normal, and overweight BMI groups (Figures 6(g) and 6(h)). This suggests that the genus *Lachnoclostridium* is associated with not only HP infection and older age, but also BMI, while *Halomonas* is only associated with HP infection and older age.

### 3.6. Analysis of the Metabolic Pathways

To characterize the significant differences in the distribution of KEGG pathways, comparisons between each two groups were performed using PiCRUSt analysis.

**Figure 7:**
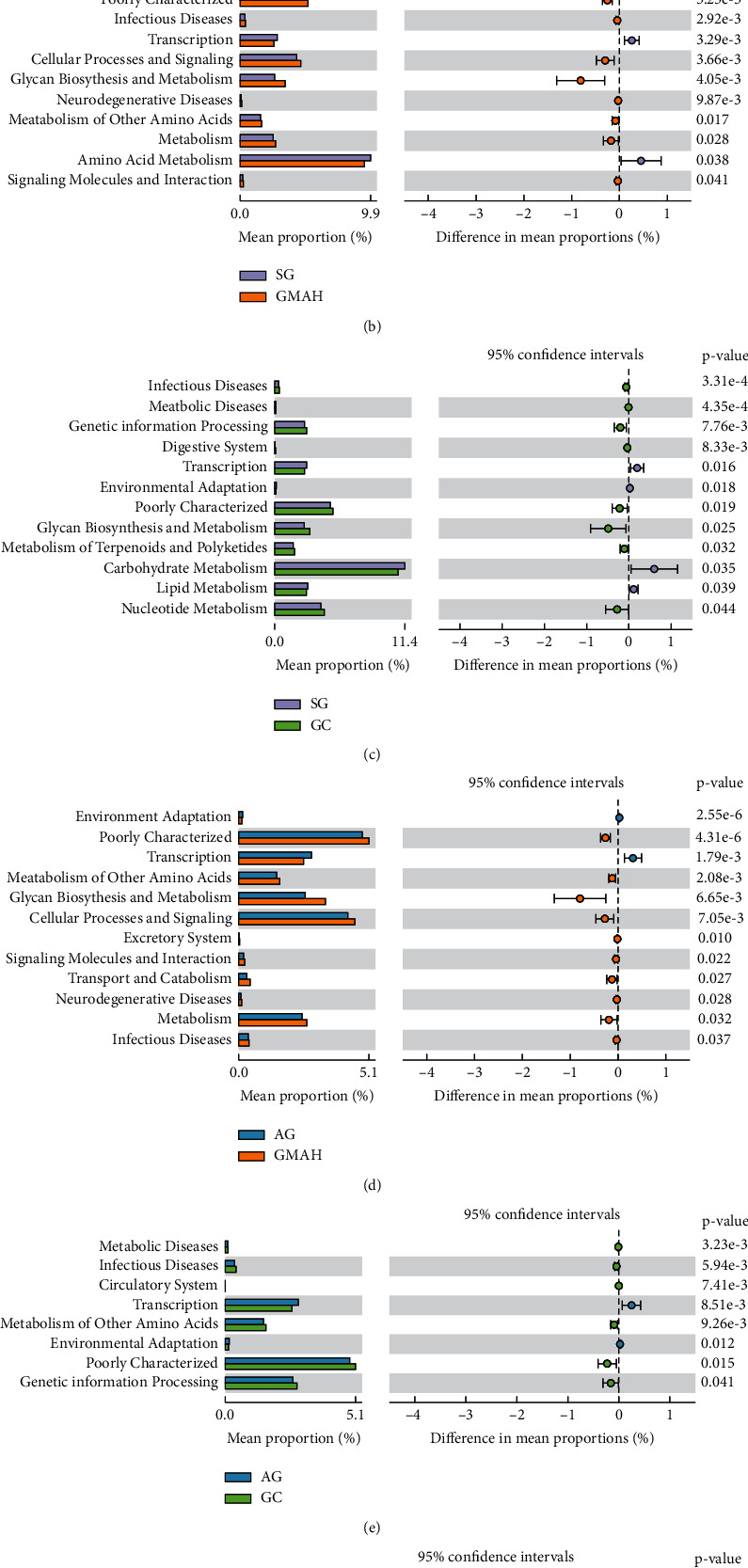
Comparison of KEGG pathways among the four groups. The pathways demonstrated significant differences between the SG and AG groups (a), the SG and GMAH groups (b), the SG and GC groups (c), the AG and GMAH groups (d), the AG and GC groups (e), and the GMAH and GC groups (f). ^*∗*^*p* < 0.05. SG: superficial gastritis; AG: atrophic gastritis; GMAH: gastric mucosal atypical hyperplasia; GC: gastric cancer.

When compared with SG, 3 KEGG pathways including Amino Acid Metabolism, Carbohydrate Metabolism, and Biosynthesis of Other Secondary Metabolites were less abundant in the AG group (Figure 7(a)). Eight KEGG pathways including Poorly Characterized, Infectious Diseases, Cellular Processes and Signaling, Glycan Biosynthesis and Metabolism, Neurodegenerative Diseases, Metabolism of Other Amino Acids, Metabolism, and Signaling Molecules and Interaction were more abundant in the GMAH group, while Environmental Adaptation, Transcription, and Amino Acid Metabolism were less abundant in the GMAH group (Figure 7(b)). Besides, 8 KEGG pathways including Infectious Diseases, Metabolic Diseases, Genetic Information Processing, Digestive System, Poorly Characterized, Glycan Biosynthesis and Metabolism, Metabolism of Terpenoids and Polyketides, and Nucleotide Metabolism were more abundant in the GC group, while Transcription, Environmental Adaptation, Carbohydrate Metabolism, and Lipid Metabolism were less abundant in the GC group (Figure 7(c)).

When compared with AG, 10 KEGG pathways including Poorly Characterized, Metabolism of Other Amino Acids, Glycan Biosynthesis and Metabolism, Cellular Processes and Signaling, Excretory System, Signaling Molecules and Interaction, Transport and Catabolism, Neurodegenerative Diseases, Metabolism, and Infectious Diseases were more abundant in the GMAH group, while Environmental Adaptation and Transcription were less abundant in the GMAH group (Figure 7(d)). Besides, 6 KEGG pathways including Metabolic Diseases, Infectious Diseases, Circulatory System, Metabolism of Other Amino Acids, Poorly Characterized, and Genetic Information Processing were more abundant in the GC group, while Transcription and Environmental Adaptation were less abundant in the GC group (Figure 7(e)). Surprisingly, the Circulatory System pathway was only significantly enriched in the GC group, instead of the GMAH group (Figure 7(f)).

## 4. Discussion

Recent studies demonstrated that the gut microbiota is a complex ecosystem with tens of trillions of microorganisms, including bacteria, fungi, archaea, parasites, and viruses [18, 19]. Gastrointestinal microflora is a risk factor associated with the occurrence of gastrointestinal tumors [20]. In recent years, the 16S rRNA sequencing technology was performed in many cancers to explore the relationship between the gut microbiota and cancer. In this study, we aim to reveal the gut microbiota differences in the different stages of GC and the potential target of noninvasive therapy in order to improve the prognosis and survival of GC patients.

We collected 7 clinical datasets from 51 subjects including 15 SG, 13 AG, 8 GMAH, and 15 GC cases and found that age, BMI, smoking/drinking status, and HP infection status were significantly different among the four groups. Besides, the age in the GMAH and GC groups was higher than the other two groups.

In addition, we collected 51 stool samples and used 16S rRNA sequencing to detect the differences of the gut microbiota composition among the four groups. We observed significant microbiome dysbiosis in different stages of GC. Firstly, Bacteroidetes, Firmicutes, and Proteobacteria were the top 3 dominant bacterial phyla among the four groups, which is consistent with the top 3 abundant bacterial phyla in different stages of GC rats [21]. Secondly, alpha diversity and beta diversity were used to assess the gut microbiota dysbiosis [22]. We found that the significant changes of the community richness and diversity were only observed at the GMAH and GC groups when compared with the SG group. This suggests that the richness or diversity of gut microbiota is significantly different at GMAH and GC stages. Moreover, beta diversity analysis was performed to investigate the structural variation of microbial communities across samples, and we found that there is significant community structure distribution among the four groups.

Thirdly, 46 species of bacteria with different abundance were identified by the LDA analysis, which included significant high abundances of 21 genera. The 8 SG-enriched genera include *Dorea*, Erysipelotrichaceae_unclassified, Ruminococcaceae_unclassified, *Fusicatenibacter*, *Faecalibacterium*, *Roseburia*, *Lachnoclostridium*, and *Butyricicoccus*. The genus *Dorea* was increased in the SG group, which is consistent with a previous study that *Dorea* was increased in gastritis [23]. Although Ruminococcaceae and *Roseburia* were reported to be increased in the chronic AG [24], in this study, these two genera were increased in the SG group instead of the AG group. The genus *Fusicatenibacter* was enriched in gastric intraepithelial neoplasia in gastric mucosal specimens [14] while we found that *Fusicatenibacter* has higher abundance in the stool specimens of SG. The 3 AG-enriched genera include *Tyzzerella_3*, *Actinomyces*, and Lachnospiraceae_unclassified. However, there is no report about the association between these three genera and AG or other stages of GC. The 4 GMAH-enriched genera include Burkholderiales_unclassified, *Peptoniphilus*, *Alloprevotella*, and *Prevotella_7*. Recently, Han et al. have shown that Burkholderiales are closely related to colorectal cancer (CRC) patients with hyperlipidemia [25]. In addition, it has been suggested that the gut microbiota Bacteroidales and Burkholderiales can modulate the immune system and play key roles in the antitumor effect of blocking CTLA-4 in CRC [26]. *Peptoniphilus* and *Prevotella_7* were enriched in the GMAH stage, but Zhang et al. reported that *Peptoniphilus* and *Prevotella* were more abundant in the GC patients than the healthy controls [10]. *Alloprevotella* showed higher abundance in the AG with gastric biopsies specimens [14], but in this study, we found that *Alloprevotella* was more abundant in the GMAH group than the other groups.

Furthermore, the 6 GC-enriched genera include *Porphyromonas*, *Scardovia*, *Halomonas*, Actinobacteria_unclassified, *Bergeyella*, and *Enterococcus*. The genera of *Porphyromonas* and *Actinobacteria* were highly enriched in the GC group, which is in accordance with previous study in GC patients [10]. What is more, *Actinobacteria* has high abundance in GC rats model [21]. *Bergeyella* was increased in the GC with gastric biopsies specimens [14], while in this study, we also found that *Bergeyella* was enriched in gut microbiota of GC patients. *Enterococcus* is one of the most common bacteria in the gastrointestinal tract [27], and infection with *Enterococcus* can cause inflammation, ROS production, and DNA damage in human gastric cancer cells [28]. In a Mongolian population, *Enterococcus* was increased in the GC patients compared to noncancer controls in gastric mucosal specimens [29]. Moreover, in this study, we found two genera *Scardovia* and *Halomonas* are enriched in the GC group, although no research study reported that these two genera are associated with GC. Therefore, further studies need to illustrate the relationship between GC and these two genera.

Besides, we found that *Enterococcus, Lachnoclostridium, Tyzzerella_3, Roseburia, Butyricicoccus*, *Dorea, Halomonas*, and Burkholderiales_unclassified were associated with HP infection. In this study, *Enterococcus* was highly abundant in the non-HP infection group, which is in accordance with the finding that *Enterococcus* was increased in the HP eradication patients during the short-term and interim follow-up [30]. The higher abundance of *Lachnoclostridium* was observed in the non-HP infection group, which is consistent with the finding that *Lachnoclostridium* was enriched in the HP infection-related gastritis patients after being treated with bismuth quadruple therapy for 14 days [31]. The abundance of *Roseburia* was lower in the HP infection group, which is consistent with the study that reported lower abundance of *Roseburia* in the HP+/CagA+ samples with gastric mucosa specimens [32]. The abundance of *Dorea* was higher in the non-HP infection group; however, a previous study indicated that the abundance of *Dorea* was reduced in the HP+ Cap polyposis after antibiotic treatment [33]. Therefore, large studies were needed to validate this issue. What is more, *Halomonas* was associated with HP infection in gastric microbiome of Indian patients [34]. However, there are few reports about the relationship between HP infection and genera *Butyricicoccus* and *Burkholderiales.*

The abundances of Erysipelotrichaceae_unclassified*, Actinomyces,* Lachnospiraceae_unclassified, and *Lachnoclostridium* genus were lower in the older age group, while the abundances of *Alloprevotella* and *Halomonas* were significantly higher in the older age group. Badal et al. reported that the abundance of Lachnospiraceae was reduced in the aging population [35]. In addition, *Eggerthella*, *Akkermansia*, *Anaerotruncus*, and *Bilophila* were positively associated with the older adults [36]. Moreover, *Lachnoclostridium* and *Prevotella_7* were associated with BMI, which are consistent with the findings of previous studies of Zhao et al. which have shown that gut microbiota *Lachnoclostridium* was more abundant in the high-fat diet rats than the normal diet rats [37]. Zhou et al. reported that *Lachnoclostridium* has significantly higher abundance in the obese polycystic ovary syndrome group when compared with the control group [38] and Zhong et al. reported that *Prevotella* was increased in nonobese individuals [39]. The evidence indicated that lower abundance of Bacteroidetes phylum and ratio of *Bacteroides*/*Prevotella* groups were related to high BMI in Brazil children [40].

Finally, yet importantly, the KEGG function prediction analysis identified several metabolic pathways associated with GC group. Interestingly, the Genetic information processing and Circulatory System pathways were more abundant in the GC group when compared with noncancer groups. Although the relationships between the abovementioned two metabolic pathways and GC are still unknown, it is suggested that the Genetic information processing and Circulatory System pathways may provide a novel understanding of the microbiome–metabolome interaction and be helpful for the therapy in GC in the near future.

Our study had several limitations. Firstly, the sample size was relatively small in each group. Secondly, healthy controls, intestinal metaplasia subjects, and early GC patients were not recruited due to the limited sources of patients in this study. Thus, it is urgent for us to enlarge our cohort and recruit more different stages of GC subjects to verify our current results in the near future.

In conclusion, our findings identified several previously unreported bacteria in the different stages of GC. Moreover, we showed that 6 genera and two metabolic pathways including Genetic information processing and Circulatory System were more abundant in the GC group than noncancer groups. These findings may contribute to the understanding of GC progression. Hence, further researches are required to elucidate the mechanisms that link gut microbiota and GC, which may provide new potential therapeutic strategies for GC.

## Figures and Tables

**Table 1:** Clinical characteristics for the 51 participates who enrolled in this study.

Items	Control (*n* = 15)	AG (*n* = 13)	GMAH (*n* = 8)	GC (*n* = 15)	*p*-value
Sex					0.521
Male	7	8	5	11	
Female	8	5	3	4	

Age	47.40 ± 12.37	45.77 ± 13.62	64.00 ± 11.83	69.60 ± 6.91	<0.0001
BMI (kg/m^2^)	25.24 ± 3.42	23.13 ± 3.53	27.04 ± 3.51	21.20 ± 3.28	0.0017

Family tumor history					0.195
Yes	2	6	3	7	
No	13	7	5	8	

Smoking					0.016
Yes	2	8	4	10	
No	13	5	4	5	

Drinking					0.02
Yes	1	5	4	9	
No	14	8	4	6	

HP infection					0.004
Yes	4	8	8	11	
No	11	5	0	4	

BMI: body mass index; HP: *Helicobacter pylori*. ^*∗*^*p* < 0.05 was considered significant.

## Data Availability

The datasets analyzed during this study are available from the BioProject database: https://www.ncbi.nlm.nih.gov/bioproject/PRJNA765747.

## Supplementary Materials

Supplementary Materials Table S1: A summary of the pyrosequencing data.
