# Supplementary material for: Gut Microbiota Dysbiosis in the Development and Progression of Gastric Cancer
Source: J Oncol. 2022 Aug 28;2022:9971619. doi: 10.1155/2022/9971619 (PMC9441395; doi:10.1155/2022/9971619)
Supplement: Supplementary Materials — Table S1: A summary of the pyrosequencing data. [file 9971619.f1.docx]

| Table S1: A summary of the pyrosequencing data. | | | | |
| --- | --- | --- | --- | --- |
|  | SG | AG | GMAH | GC |
| OTUs | 2414 | 2860 | 1166 | 2531 |
| Phylum | 11 | 12 | 9 | 14 |
| Class | 24 | 25 | 20 | 28 |
| Order | 37 | 40 | 34 | 50 |
| Family | 67 | 70 | 64 | 86 |
| Genus | 136 | 142 | 134 | 180 |

OUT, operational taxonomic unit; SG, superficial gastritis; AG, atrophic gastritis; GMAH, gastric mucosal atypical hyperplasia; GC, gastric cancer.
